# Using Routinely Reported Tuberculosis Genotyping and Surveillance Data to Predict Tuberculosis Outbreaks

**DOI:** 10.1371/journal.pone.0048754

**Published:** 2012-11-07

**Authors:** Sandy P. Althomsons, J. Steven Kammerer, Nong Shang, Thomas R. Navin

**Affiliations:** 1 Centers for Disease Control and Prevention, Division of Tuberculosis Elimination, Atlanta, Georgia, United States of America; 2 Northrop Grumman Corporation, Centers for Disease Control and Prevention Programs, Atlanta, Georgia, United States of America; McGill University, Canada

## Abstract

We combined routinely reported tuberculosis (TB) patient characteristics with genotyping data and measures of geospatial concentration to predict which small clusters (i.e., consisting of only

3 TB patients) in the United States were most likely to become outbreaks of at least 6 TB cases. Of 146 clusters analyzed, 16 (11.0%) grew into outbreaks. Clusters most likely to become outbreaks were those in which at least 1 of the first 3 patients reported homelessness or excess alcohol or illicit drug use or was incarcerated at the time of TB diagnosis and in which the cluster grew rapidly (i.e., the third case was diagnosed within 5.3 months of the first case). Of 17 clusters with these characteristics and therefore considered high risk, 9 (53%) became outbreaks. This retrospective cohort analysis of clusters in the United States suggests that routinely reported data may identify small clusters that are likely to become outbreaks and which are therefore candidates for intensified contact investigations.

## Introduction

Centers for Disease Control and Prevention (CDC) guidelines recommend that close contacts of persons with infectious tuberculosis (TB) be investigated to identify persons who might have active TB disease, as well as those who might have TB infection that has not yet progressed to active disease [1]. Treatment of persons with latent TB infection can prevent the development of active TB; unsuccessful or incomplete contact investigations may result in additional cases of TB and further TB transmission [2],[3],[4]. A review of 27 TB outbreaks investigated by CDC found that the most common intervention to control an outbreak was an intensified contact investigation to identify previously missed contacts of patients and prioritize them for evaluation and treatment, based on risk for progression to disease [5]. State and local health departments' investigation reports also recommend contact investigations as a response to TB outbreaks [6],[7],[8].

An intensified contact investigation conducted while the number of patients is small might be more effective and cost less than after a large outbreak has developed. Although TB outbreaks are not common (all 27 outbreaks investigated by CDC occurred over a 7-year period), they can be labor-intensive and expensive. A recent outbreak in a homeless shelter reportedly cost an additional $200,000 above routine health care services [9]. If public health officials had substantially more resources than are currently available, they could conduct intensive contact investigations of all geographically concentrated TB cases. At a time of reduced resources, however, we propose an approach that uses routinely collected data to formulate an algorithm that would predict which clusters of cases are most likely to become outbreaks. To be most cost effective, early interventions should distinguish between groups of cases at high risk for becoming outbreaks from groups at low risk.

Routine genotyping of *Mycobacterium tuberculosis* from patients in the United States identifies genotype clusters and provides insights into the location, timing, and circumstances of TB transmission [10],[11]. Previous studies have identified factors that predict growth of TB genotype clusters. New clusters detected in New York City grew more rapidly if both the first 2 patients had sputum smears positive for acid-fast bacilli and cavitary lesions on chest radiographs [12]. In the Netherlands, rapid initial growth (defined as <3 months between the diagnosis of the first and the second cases) was associated with the highest odds for cluster growth; other significant predictors were age <35 years, urban residence, and both patients having been born in sub-Saharan Africa [13]. We analyzed TB genotyping, geospatial, and patient data routinely reported to CDC to determine factors that best predicted which small (i.e., only 3 patients) incident clusters were most likely to become outbreaks.

## Methods

We included TB cases that had valid genotyping data and were reported by the 50 states and Washington, D.C., to the CDC National Tuberculosis Surveillance System during 2004–2010 [14], the latest genotyping data available at the time of this analysis. Genotyping data were obtained from the CDC National Tuberculosis Genotyping Service by methods described elsewhere [10]. *M. tuberculosis* culture isolates were analyzed to determine spoligotype and 12-locus mycobacterial interspersed repetitive units-variable number tandem repeats (MIRU-VNTR) pattern. Two patients were considered to have matching genotypes if their isolates had indistinguishable spoligotype and MIRU-VNTR patterns. A genotype cluster was defined as 2 or more TB patients with matching genotypes in the same geographic area.

The statistical program SaTScan, version 9.1.0, was used to identify spatially concentrated clusters of TB cases with a specific genotype during 2006–2010; we used residential zip code as the geographic unit of measurement [15]. We applied the discrete Poisson probability model, using all culture-positive TB cases as the background population. SaTScan uses the spatial scan statistic, based on the log-likelihood ratio (LLR), to determine spatial concentration of cases in a cluster. SaTScan identified significant clusters with the smallest p-value first. Additional cases not yet assigned to a cluster were evaluated to identify additional clusters. Parameters were set so that individual cases were allowed membership into only 1 cluster. Clusters with both significant (p<0.05) and nonsignificant concentrations were included in the cohort. We set a cluster's radius to be no more than 50 kilometers, as previous analyses demonstrated that setting a maximum of 100 kilometers produces the same clusters, while a 20-kilometer radius may split clusters [*11*]. To focus on incident rather than endemic clusters (i.e., those present over a long period of time) [16], we restricted our analysis to new clusters, defined as those in which the initial case occurred during 2006–2008 and was preceded by a 24-month period of no reported cases. Routine genotyping was not initiated in all areas at the same time, and in 2006, national genotyping coverage, defined as the proportion of culture-positive cases with a reported genotype in the National Tuberculosis Genotyping Service, was 70%. When an area first begins genotyping, all clusters in the area will appear to be new. To avoid inclusion of endemic clusters as newly emerging strains from areas with incomplete genotyping coverage, we excluded clusters if the county with the most cases had <75% annual genotype coverage. If a cluster had the majority of its cases in a county that did not meet this criterion, the entire cluster, not just the cases, was excluded.

To ensure that clusters had an equal chance (i.e., an equal time period) to become outbreaks, we established a standardized observation period of 24 months after the third case. The 24-month follow-up period was derived from an analysis of the time between the third and sixth cases (or the last case, if the cluster did not reach 6 cases by 2010). The longest time interval observed was 23.9 months (data not shown). This approach identified 148 new clusters of at least 3 cases that could be observed for 24 months.

Although no standard definition of a TB outbreak exists, for the purposes of this analysis, we defined an outbreak as a cluster that grew from 3 to at least 6 cases during the observation period, in which at least two of the cases could be linked epidemiologically (i.e., had spent time in the same place when at least one of them was contagious), and in which the cluster was confirmed to be an outbreak by local public health officials (usually state as well as county TB control officers). Time intervals between dates of diagnoses were calculated based on the earliest of 3 possible dates (i.e., the date a patient specimen was collected for drug susceptibility testing, the date TB treatment was initiated, or the date the patient was counted as a verified TB case). We used the rate of initial cluster growth as a predictive variable; we considered the times between diagnosis of the first and second case, between the first and the third case, and between the second and the third case. We used SaTScan to determine which clusters were significantly concentrated (p<0.05) at the time of the third case. (SaTScan could not define significance for 3 clusters.)

Other predictive variables were based on patient characteristics reported to the National Tuberculosis Surveillance System, described elsewhere [14],[17]. The unit of analysis was the cluster. A cluster was considered “exposed” by a characteristic if any 1 of the first 3 patients had that characteristic. For brevity in this report, we refer to patients who reported homelessness or excess alcohol use or illicit drug use in the 12 months before diagnosis, or who reported being incarcerated at the time of TB diagnosis as being “homeless, incarcerated, or drug or alcohol users.” Clusters with at least 1 of the first 3 patients who reported any of these conditions are described as “marginalized” in this context. Socioeconomic measures for crowding, education, and unemployment were derived from the 2000 U.S. Census; median values were calculated for all zip codes. A cluster was considered “exposed” if the zip code with the most cases had a value above the median. For clusters from multiple zip codes with equal numbers of cases, one zip code was randomly selected. The influence of genotype lineages was assessed based on spoligotyping of TB in the United States; lineages include *M. bovis* as well as the subgroups of Indo-Oceanic, Euro-American, East Asian, and East-African Indian [18],[19]. (*M. Africanum* was not identified in the cohort.) Univariate analysis was performed to describe a cluster's risk for becoming an outbreak. We used SAS 9.2 (SAS, Cary, NC, USA) to calculate relative risks and 95% confidence intervals (CI).

SAS JMP 9.0.1 was used for the decision-tree analysis, based on recursive partitioning, to determine which combination of variables best predicted clusters that became outbreaks. JMP compares possible binary partitions based on the LogWorth statistic, which is calculated as -log10 (adjusted p-value), where the adjusted p-value takes into account the number of different ways partitions can occur for each variable [20]. JMP determines the partition that best predicts the outcome of interest for both continuous and categorical variables. When the decision-tree analysis identified a partition that resulted in a node with fewer than 20 clusters, we stopped the partitioning.

Approval by an institutional review board was not required because data were collected and analyzed for this project as part of routine TB surveillance, and the project was therefore not considered research involving human subjects.

## Results

### Determining which clusters grew to become outbreaks

**Figure 1:**
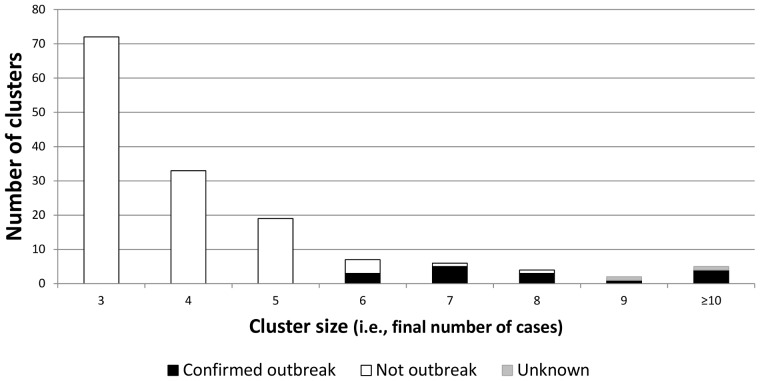
Distribution of 148 cohort clusters, by number of cases and outcome. Cohort composed of incident tuberculosis genotype clusters of 3 or more cases identified by SaTScan from 2006 to 2010, meeting inclusion criteria. Clusters with 10 or more cases are grouped into one bar. Outcome of the cluster could be confirmed as an outbreak, confirmed as not an outbreak, or unable to be confirmed (uncertain).

**Figure 2:**
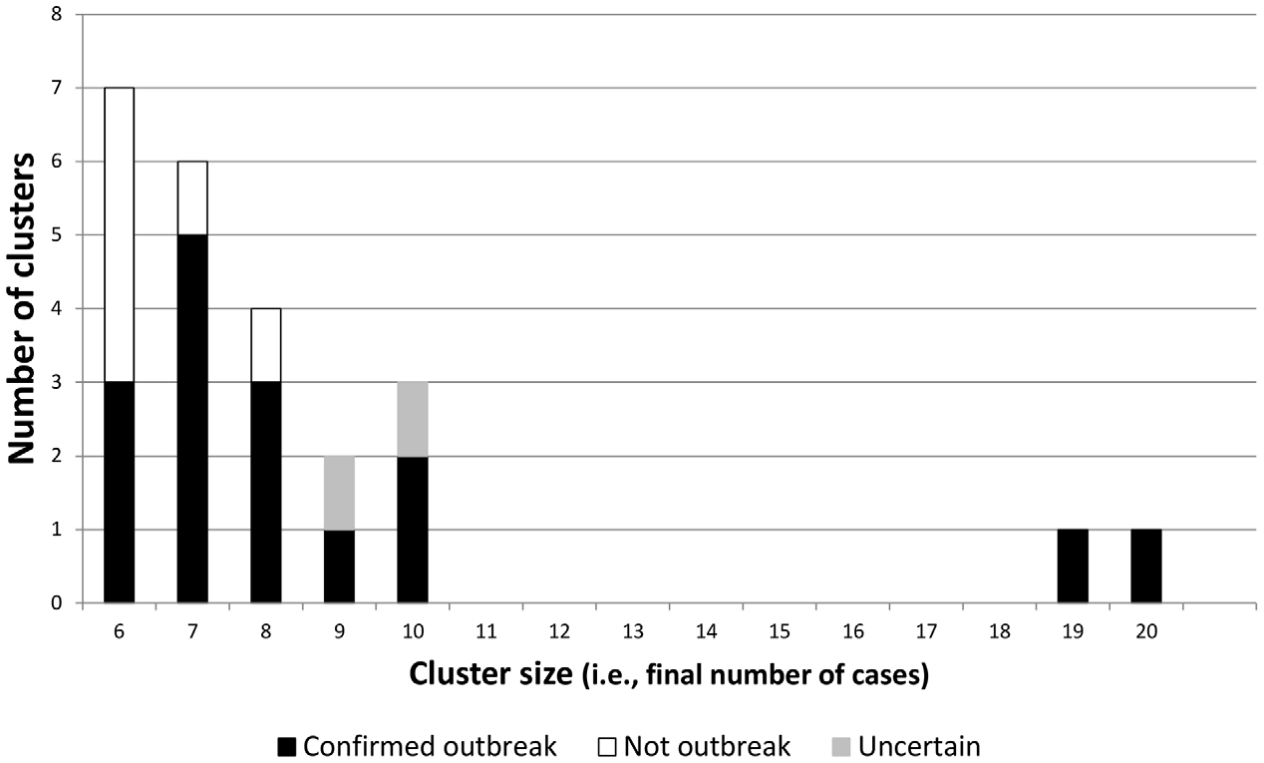
Higher resolution of Figure 1. Subcohort of 24 clusters of 6 or more cases, without grouping clusters with 10 or more cases into one bar.

148 clusters met our inclusion criteria (identified by SaTScan with an initial case from 2006 through 2008, in which the county with most cases had >75% annual genotype coverage and a 24-month observation period followed the 3^rd^ case). Of these, 24 (16.2%) grew to at least 6 cases within 24 months. Local public health officials reported that 16 (66.7%) of the 24 clusters were known to be outbreaks, 6 were not, and 2 could not be classified with certainty. Both clusters with uncertain outcomes were considered possible outbreaks by local officials, but neither was investigated intensively at the time the initial cases were reported, and no epidemiologic links could be verified at the time of our inquiry; these two clusters were excluded from our univariate and decision-tree analysis. Our final analytic cohort included 146 new clusters that were from 26 states distributed across the United States, began during 2006–2008, contained at least 3 TB cases, could be observed for 24 months after the third case, and could be classified as either an outbreak or not an outbreak by the end of our 24-month observation period (Figure 1, Figure 2).

The two largest outbreaks predominantly involved homeless persons and had grown to 19 and 20 cases, respectively, by the end of our observation period. All patients from one outbreak were black non-Hispanic and born in the United States; for the other, 11 patients were Hispanic and most were born in Mexico. In another 3 outbreaks, at least half the patients were homeless. Two outbreaks were centered in correctional institutions. Of the remaining outbreaks, the predominant characteristics were excess alcohol use (3 outbreaks), substance abuse (3), and a combination of excess alcohol and substance use (2). The final outbreak involved 7 persons born in the U.S.- affiliated Pacific Islands or with parents born there; none reported homelessness, excess alcohol use or illicit drug use, or were incarcerated at the time of TB diagnosis. Of the 16 outbreaks, 14 were known to be outbreaks by local officials before our inquiry, but 2 clusters were designated as outbreaks only after investigations conducted as a consequence of our inquiry. All 16 confirmed outbreaks had a statistically significant SaTScan result at the end of the 24-month observation period, meaning cases in these clusters were measurably more geospatially concentrated than were cases of the same genotype in the rest of the country.

### Relative risks of outbreak predictors

Although homelessness, excess alcohol use, illicit drug use, and incarceration as individual predictors were each significantly associated with clusters that became outbreaks, clusters in which at least 1 of the first 3 patients reported any one of these social risk factors (i.e., a “marginalized” cluster) had the highest relative risk for becoming an outbreak (RR = 17.7, 95% CI 2.4, 130.4; Table 1). We also looked at different combinations of these characteristics, e.g., homelessness, excess alcohol use, or illicit drug use without consideration of incarceration status. By far the most discriminatory factor was if any of the 4 conditions was reported, followed by whether a patient reported homelessness, excess alcohol use, or illicit drug use, and then by whether a patient reported either homelessness or excess alcohol use, or both (Table 1).

**Table 1:** Patient and cluster characteristics of tuberculosis (TB) genotype clusters and associated risk of the cluster becoming an outbreak within 24 months after diagnosis of the 3^rd^ patient.

Cluster characteristic* (n = 146)	Cluster outcomes, n (%)		Relative risk in predicting confirmed outbreaks
Patient risk factors	Confirmed outbreak (n = 16)	Not an outbreak (n = 130)	RR (95% CI)
Homeless or excess alcohol use or illicit drug use or incarceration at diagnosis	15 (93.8)	52 (40.0)	17.7 (2.4, 130.4)
Homeless or excess alcohol use or illicit drug use	14 (87.5)	50 (38.5)	9.0 (2.1, 38.0)
Illicit drug use	12 (75)	35 (26.9)	6.3 (2.2, 18.6)
Excess alcohol use	11 (68.8)	33 (25.4)	5.1 (1.9, 13.8)
Homeless or excess alcohol use	11 (68.8)	39 (30.0)	4.2 (1.6, 11.5)
Homeless and excess alcohol use	6 (37.5)	14 (10.8)	3.8 (1.5, 9.3)
Incarceration	5 (31.3)	12 (9.2)	3.4 (1.4, 8.7)
Homeless	6 (37.5)	20 (15.4)	2.8 (1.1, 6.9)
HIV infection	4 (25.0)	21 (16.2)	1.6 (0.6, 4.6)
Unemployed	12 (75.0)	104 (80.0)	0.8 (0.3, 2.2)
Health care worker	0 (0)	10 (7.7)	n/a
**Disease factors**			
Sputum AFB smear + and cavitary lesions^1^	13 (81.3)	73 (56.2)	3.0 (0.9, 10.2)
Cavitary lesions	13 (81.3)	75 (57.7)	2.9 (0.9, 9.6)
Sputum AFB smear positive	15 (93.8)	117 (90)	1.6 (0.2, 11.2)
Multidrug-resistant TB^2^	1 (6.3)	5 (3.9)	1.6 (0.2, 9.9)
History of previous TB	1 (6.3)	12 (9.2)	0.7 (0.1, 4.8)
INH drug resistance	1 (6.3)	18 (13.9)	0.5 (0.06, 3.2)
**Patient demographics**			
American Indian or Alaska Native race	2 (12.5)	2 (1.5)	5.1 (1.7, 15.2)
Black, non-Hispanic	9 (56.3)	54 (41.5)	1.7 (0.7, 4.3)
Age<15 years	3 (18.8)	16 (12.3)	1.5 (0.5, 4.9)
White, non-Hispanic	3 (18.8)	25 (19.2)	1.0 (0.3, 3.2)
Hispanic	6 (37.5)	67 (51.5)	0.6 (0.2, 1.6)
Asian, non-Hispanic	1 (6.3)	38 (29.2)	0.2 (0.03, 1.3)
Foreign-Born	5 (31.3)	107 (82.3)	0.1 (0.05, 0.4)
Less than 2 years in US	1 (33.3))	38 (44.7)	0.6 (0.1, 6.7)
SES^3^ – crowded household	7 (43.8)	66 (50.8)	0.8 (0.3, 2.0)
SES^3^ – less than high school education	8 (50.0)	58 (44.6)	1.2 (0.5, 3.1)
SES^3^ – unemployment	6 (37.5)	62 (47.9)	0.7 (0.3, 1.8)
SES^3^ – some college education	9 (56.3)	71 (54.6)	1.1 (0.4, 2.7)
Genotype lineage *M.bovis*	0 (0)	5 (3.9)	n/a
Genotype lineage subgroup Indo-Oceanic	0 (0)	15 (11.5)	n/a
Genotype lineage subgroup Euro-American	12 (75.0)	86 (66.2)	0.7 (0.2, 2.0)
Genotype lineage subgroup East Asian	4 (25.0)	15 (11.5)	0.4 (0.2, 1.2)
Genotype lineage subgroup East-African Indian	0 (0)	9 (6.9)	n/a
Male	16 (100.0)	116 (89.2)	n/a
Native Hawaiian or other Pacific Islander race	0 (0)	1 (0.7)	n/a
**Cluster characteristics**			
Initial cluster growth rate (time between patients)			
1st and 3rd patient <5.3 months	9 (56.3)	33 (25.4)	3.2 (1.3,8.0)
1st and 2nd patient <4.4 months	12 (75.0)	59 (45.4)	3.2 (1.1, 9.4)
2nd and 3rd patient <0.9 months	7 (43.8)	26 (20.0)	2.7 (1.1, 6.6)
Significant log likelihood ratio at 3rd case	13 (81.3)	67 (52.3)	3.5 (1.0, 11.6)

* One or more of 1^st^ three patients had characteristic.

1 Patient had acid fast bacilli smear-positive sputum specimens and abnormal chest radiograph results with evidence of cavities.

2 Patient had isoniazid (INH) and rifampicin (RIF) drug resistance reported in initial susceptibility drug test.

3 Median values for socioeconomic measures were derived from the 2000 U.S. Census for all zip codes. A cluster was considered “exposed” if the zip code with the most cases had a value above the median.

Clusters in which at least 1 of the first 3 patients was born outside the United States were significantly less likely to become outbreaks (RR = 0.1, 95% CI 0.05, 0.4). The only other significant demographic risk factor was the presence of an American Indian or Alaska Native among the first 3 patients. Each of our 3 measurements of initial cluster growth rate was a significant predictor, but the optimal measure was if the third case occurred within 5.3 months of the first patient (RR = 3.2, 95% CI 1.3, 8.0). Finally, a spatially concentrated cluster (significant SaTScan result at third patient) was also significantly associated with the cluster becoming an outbreak (RR = 3.5, 95% CI 1.0, 11.6). Neither socioeconomic measures based on Census data of residential zip codes nor genotype lineage was associated with clusters that became outbreaks (Table 1).

### Decision-tree analysis to predict outbreaks

**Figure 3:**
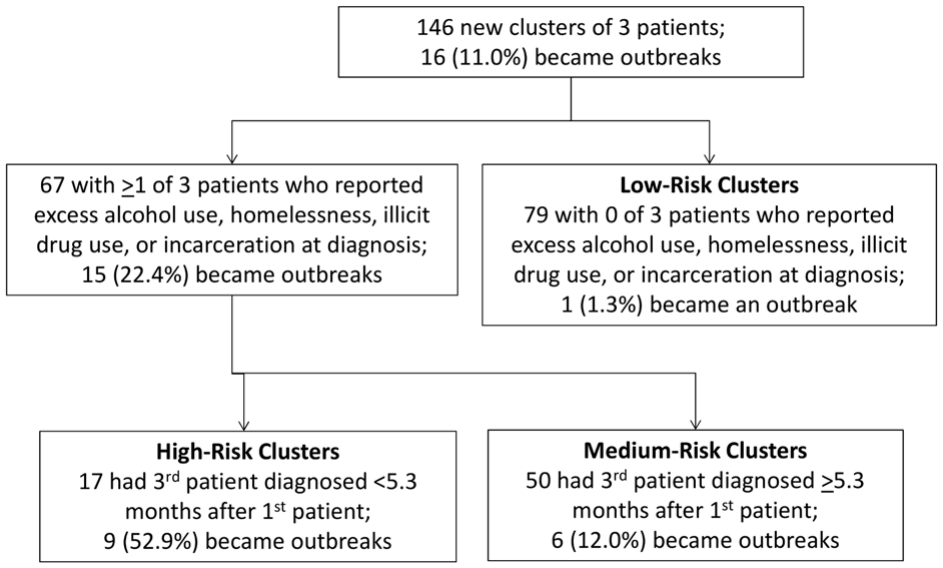
Algorithm based on decision-tree analysis for predicting TB outbreaks. Algorithm based on data available at time the TB cluster contained 3 cases. Decision-tree analysis categorizes clusters in high-, medium-, or low-risk groups. Clusters in the high-risk group are considered of greatest priority for early interventions, such as intensive contact investigations. Although clusters in medium- and low-risk groups may not be considered highest priority when they have 3 cases, they can be re-evaluated should additional cases occur.

The most discriminatory variable identified by JMP was also the variable with the highest relative risk—specifically, if the cluster was marginalized (Figure 3). Sixty-seven (45.9%) of the 146 clusters with known outcomes were marginalized; 15 (22.4%) of these became outbreaks. Of the other 79 clusters (i.e., clusters with no social risk factors among the first 3 patients), only 1 (1.3%) became an outbreak.

Of the 67 marginalized clusters, the most discriminatory variable for the next partition was rapid initial growth. Available measures of initial growth included time between diagnoses of the first and second, first and third, and second and third cases. JMP determined the optimal partition was 5.3 months between the first and third case. Of 17 clusters characterized as marginalized and with rapid initial growth, 9 (52.9%) became outbreaks. Of the 50 clusters that were marginalized but had slow initial growth (i.e., 5.3 months or more to third patient), 6 (12.0%) became outbreaks. Our outbreak prediction algorithm, which classified clusters based on social risk factors among the first 3 patients and rapid initial growth as being at high risk of becoming outbreaks, had a sensitivity of 56.3% and a specificity of 93.8%.

### Sensitivity analysis of decision-tree results

We analyzed alternative criteria for social risk factor characteristics among the first 3 patients. In rank order, the most discriminatory combination was if any of the first 3 patients had any social risk factor characteristic (LogWorth = 4.9), followed closely by whether the first or second patient had any social risk factor characteristic (LogWorth = 4.5), and then by whether the first patient had any social risk factor characteristic (LogWorth = 4.4). Less discriminatory combinations were those in which 2 of the 3 patients (LogWorth = 3.2) or both the first and second patients (LogWorth = 1.2) or all 3 patients (LogWorth = 0.5) had any social risk factor characteristic. Regardless of whether the 2 uncertain clusters were reclassified as either outbreaks or not outbreaks, JMP determined the same predictive variables (data not shown).

## Discussion

Our retrospective cohort analysis of TB genotype clusters showed that TB patient data and genotyping results routinely reported to CDC could be used to identify certain small clusters that were likely to become outbreaks. The two most important factors that predicted outbreaks were the presence of at least 1 patient who reported homelessness, excess alcohol use, illicit drug use, or incarceration, and rapid initial cluster growth (i.e., <5.3 months between diagnosis of the first and third case). These data suggest that if recent transmission of TB occurs among patients with the above-described social risk factors, the risk of a TB outbreak increases.

Univariate analysis produced a table of risk ratios for each predictor variable considered. Traditionally, multivariate logistic regression analysis would be used to demonstrate the adjusted contribution of significant variables toward an outcome. Instead, we used JMP for decision-tree analysis, which searched for the most important combinations of predictor variables through dichotomous splits of the data at each decision node [21]. Rather than a list of significant predictor variables, JMP determined which combinations of cluster characteristics best described those at highest risk of becoming outbreaks and reported the frequency of outbreaks for the combination of variables. We can interpret this as the percentage of high-risk outbreaks that might have been prevented. This information can be readily applied by public health officials to determine which clusters warrant intensive contact investigations, while they are still small [22].

Kik et al. also found that initial cluster growth predicted which small clusters grew to become large clusters [9]. They investigated time between the first and the second case and found the most predictive interval was <3 months. We found time to the third case to be the most predictive measure, although we also found that <4.4 months from the first to the second case was associated with clusters that became outbreaks (Table 1). We applied the same methodology described here to clusters of only 2 cases. The same predictors, notably marginalized clusters and rapid initial growth, were identified, but only 19.7% (20 of 66 clusters) with these predictive risk factors became outbreaks (data not shown). While the number of outbreaks identified at the second case would be greater, considerably more clusters would have to be investigated, and the cost per outbreak prevented would be higher.

Driver et al. [8] found that acid-fast bacilli smear-positive disease in the presence of cavitary lesions on chest radiographs was associated with cluster growth. In our analysis, this combination of factors had an elevated relative risk (3.0), but was not statistically significant (Table 1). Smear-positivity, cavitation on chest radiograph, and HIV status, which have been commonly associated with TB transmission and TB disease progression [23],[24],[25],[26], surprisingly were not found to be predictive in our decision-tree analysis. We hypothesize this finding may be due to greater and earlier attention given to these cases, which may have prevented further transmission. Clusters in which a foreign-born individual was among the first 3 cases were less likely to become an outbreak, an interesting finding considering the larger burden of TB among foreign-born persons within the United States [27]. Still, this supports other studies documenting less TB transmission among foreign-born than US-born persons [28],[29].

### Limitations

Several limitations to our analysis should be considered. We conducted a retrospective cohort analysis, and predictions based on historical data may not translate into accurate prospective predictions. Furthermore, our cohort met specific inclusion criteria that excluded many clusters. Our conclusions assumed that genotyping results and patient characteristics are known when cases are diagnosed. In practice, there are delays from when a patient is suspected of having TB to when data are available for analysis [30]. Improved timeliness of reporting this information will improve the impact of outbreak prediction algorithms. Even if an algorithm can accurately predict which clusters will become outbreaks, the value of such a prediction is predicated on the assumption that an early intervention can prevent future TB cases. The effectiveness and the cost effectiveness of early interventions remain to be determined.

National TB surveillance data in the United States capture incarceration only at the time of diagnosis but not past incarceration. Documented analysis of social risk factor data quality in California demonstrates >90% concordance with medical records [31], but self-reporting of social risk factors may be under disclosed due to social stigma [5]. Furthermore, there is no national surveillance for TB outbreaks in the United States. In fact, there is no standard definition for what constitutes a TB outbreak. Our definition of an outbreak as necessarily consisting of 6 patients was arbitrary, and local officials may consider clusters of 5 patients (or even smaller) to be outbreaks. While we can accurately report the classification of the clusters SaTScan detected, we have no way of knowing how many outbreaks SaTScan did not detect.

We were unable to assess the contact investigations that might have occurred around the initial patients of a cluster or whether an intensified contact investigation might have been conducted in the early stages of a cluster, when clustered cases might have been identified by local public health officials. Early, intensive contact investigations may have occurred for some of our high-risk clusters, and in some instances outbreaks might have been prevented. Such interventions might explain, for example, some of the 8 clusters in the “High-Risk Clusters” box in Figure 3 that did not become outbreaks. If so, the proportion of high-risk clusters that would, without any intervention, become outbreaks would be higher, increasing the sensitivity of our outbreak prediction algorithm.

The sensitivity of our prediction model (56.3%; 9 of 16 outbreaks predicted) could underestimate the true value of our algorithm. Although our algorithm incorrectly classified 7 of 16 outbreaks as not being at high risk for becoming an outbreak when the cluster had 3 patients, public health officials would have the chance to reconsider that risk when the cluster grew to 4 patients. The general approach we have described here could be used in the future to determine risk factors for clusters of 2, 4, or 5 patients becoming outbreaks. Finally, our analysis was limited by the genotyping methodology on which it was based (i.e., spoligotype and 12-locus MIRU-VNTR). Since 2009, CDC has analyzed all isolates by spoligotype and an expanded panel of 24 MIRU-VNTR loci [10]. Because 24-locus MIRU-VNTR, as well as whole genome sequencing, is more discriminatory than 12-locus MIRU-VNTR [32],[33], we expect that future outbreak prediction algorithms will have better accuracy. By having sufficient time to calculate background rates, we also expect that additional data will allow us to address outbreaks that occur within “endemic” strains.

TB outbreaks are costly, and the bigger they grow, the more resource-intensive they become. Our outbreak prediction algorithm could be used to identify small clusters that are candidates for intensive contact investigations to prevent outbreaks. Because our algorithm is based on data routinely reported to CDC, alerts can be generated automatically to ensure high-risk clusters are brought to the attention of public health officials as candidates for intensive contact investigations. As more data are collected, we may refine this algorithm and produce risk factors at different sizes of small clusters, including clusters of 2 cases. Still, this is the first quantitative measure of early risk factors associated with TB outbreaks in the United States. Using this information to prioritize clusters for resource expenditure may improve outbreak interventions.
